# A randomized, double-blind, controlled, parallel group study with amustaline/glutathione pathogen reduced red blood cells in regions at potential risk for Zika virus transfusion-transmitted infections (RedeS Study)—protocol for a phase 3 clinical trial

**DOI:** 10.1186/s13063-026-09808-y

**Published:** 2026-05-26

**Authors:** Edgardo F. Cartagena Ayala, Lumen Vera Colon, Yesim Aydinok, Bolívar Arboleda-Osorio, Arthur Bracey, Ross Fasano, Marianne Yee, Ravindra Sarode, Abba C. Zubair, Frank Nizzi, Sanjay Shah, Edward L. Snyder, Angela Treml, Yan Zheng, Clifford Takemoto, Jeffrey Green, Bryon Jackson, John P. Pitman, Thomas J. Gniadek, Kathy Liu, Stanley Bentow, Laurence Corash, Nina Mufti, Richard J. Benjamin

**Affiliations:** 1 Menonita Caguas, Caguas, Puerto Rico; 2 Aibonito, Puerto Rico; 3 Ege University Hospital, Izmir, Turkey; 4 HIMA Caguas, Caguas, Puerto Rico; 5 Baylor St. Lukes Hospital, Houston, TX USA; 6 Emory University, Atlanta, GA USA; 7 UT Southwestern, Dallas, TX USA; 8 Mayo Clinic, Jacksonville, FL USA; 9 Phoenix Children’s Hospital, Phoenix, AZ USA; 10 Yale University, New Haven, CT USA; 11 Versiti, Milwaukee, WI, USA; 12 St. Jude’s Children’s Hospital, Memphis, TN USA; 13 Virginia Commonwealth University, Richmond, VA USA; 14 University of Maryland, Baltimore, MD USA; 15 Cerus Corporation, 1220 Concord Ave, Concord, CA 94520 USA

**Keywords:** Amustaline/GSH, INTERCEPT, Anemia, Pathogen-reduction, Transfusion-transmitted infections, Randomized controlled trial, Sickle cell disease, Red cell exchange

## Abstract

**Background:**

Red blood cell (RBC) transfusion may be lifesaving, however transfusion-transmitted infections (TTI) and transfusion-associated graft-versus-host disease (TA-GVHD) still pose a threat despite current donor selection and testing requirements. Amustaline (S-303)/glutathione (GSH) pathogen reduction (PR) is designed to inactivate infectious microbes and leukocytes in RBCs. The RedeS study evaluates the safety and efficacy of amustaline/GSH PR-RBCs compared to conventional RBCs in a broad spectrum of patients requiring acute and/or repeated RBC transfusion support.

**Methods:**

RedeS is a Phase 3, prospective, multi-center, randomized, double-blind, active-controlled, parallel-design study, with a 6-month extension option to evaluate patients requiring repeated transfusions including red cell exchange (RCE). Eligible subjects will be stratified according to site, baseline bleeding status and participation in the extension period. The study arms include a transfusion period of 28-days for anemia with any bleeding or non-bleeding patients or a 28-day + 6-month extension for non-bleeding patients with chronic anemia requiring repeated simple transfusions, or for patients with sickle cell disease (SCD) expected to receive three RCE procedures.

The study will seek to enroll 600–800 subjects in the overall safety analysis, including ~ 140 repeatedly-transfused subjects in the 6-month extension including up to 30 subjects receiving three RCE procedures. The primary safety endpoints are the proportion of patients with any treatment-emergent adverse events (TEAEs) related to study RBC transfusion through 28 days after the last study transfusion, and the proportion of patients with treatment-emergent antibodies with confirmed specificity to PR-RBCs. The primary efficacy endpoint is the hemoglobin (Hb) increment in subjects who are non-bleeding at baseline, adjusted for the grams of Hb transfused and averaged over multiple transfusions. With 352 evaluable transfused non-bleeding subjects (176 per treatment group) from either arm, the study will have 80% power to demonstrate non-inferiority, defined as a Hb increment treatment difference of no more than 15% of the Control group mean, assuming a Control arm coefficient of variation of 50%.

**Discussion:**

RedeS will characterize the safety of amustaline/GSH PR-RBCs and is designed to demonstrate the non-inferiority of PR-RBCs to conventional RBCs with respect to Hb increment, a common pragmatic clinical assessment of RBC transfusion effectiveness.

**Trial registration:**

A Randomized, Double-Blind, Controlled, Parallel Group Study with the INTERCEPT Blood System for red blood cells in Regions at Potential Risk for Zika Virus Transfusion-Transmitted Infections (RedeS Study). ClinicalTrials.gov ID NCT03037164. Registered on 27 January 2017 at https://clinicaltrials.gov/ct2/show/NCT03037164.

**Supplementary Information:**

The online version contains supplementary material available at 10.1186/s13063-026-09808-y.

## Introduction

**Fig. 1:**
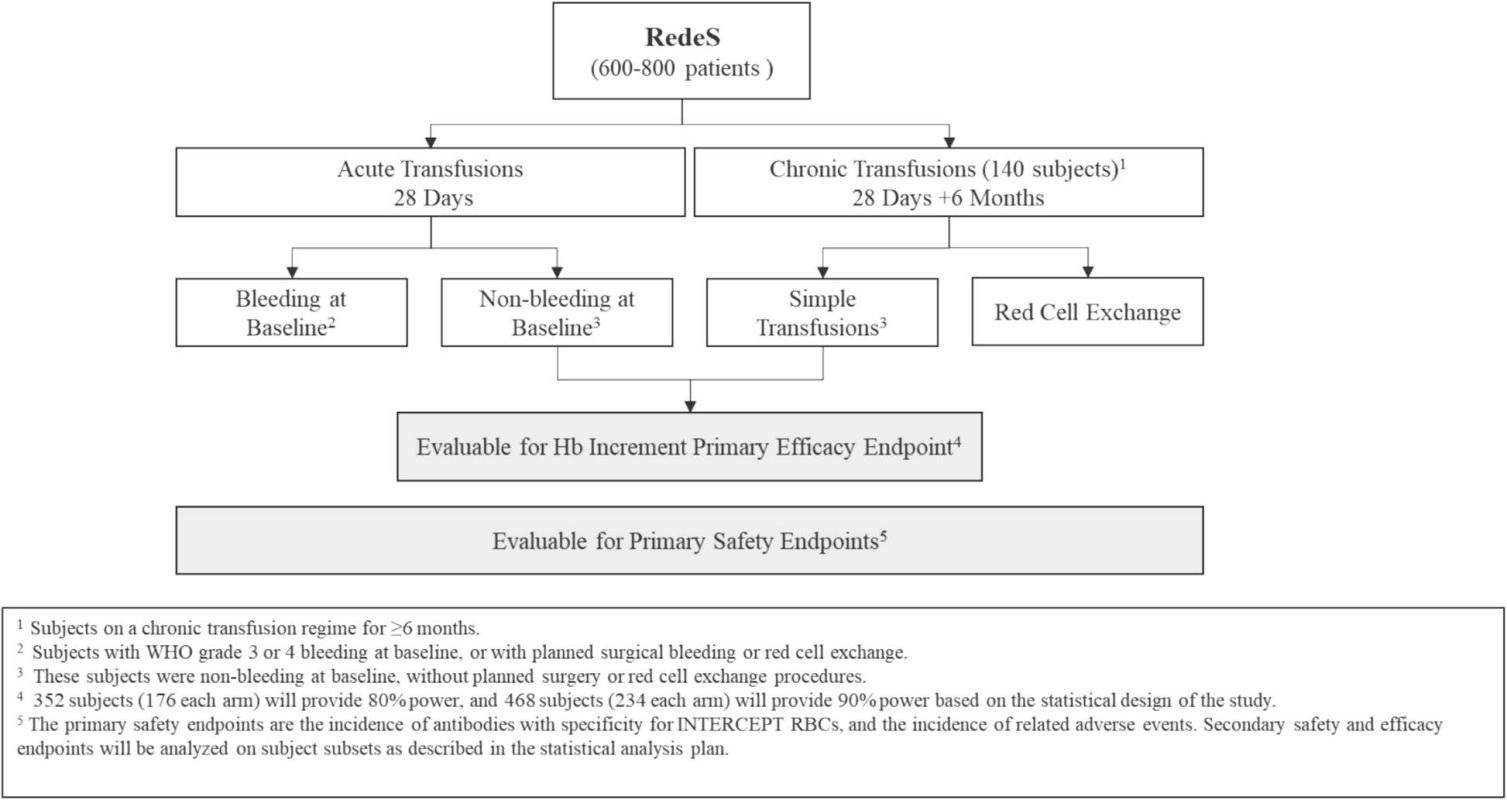
Sub-populations for analysis

The RedeS study was initially designed to evaluate the safety and efficacy of amustaline/glutathione (GSH) pathogen reduced (PR) red blood cells (RBCs) and to reduce the risk of transfusion-transmitted infections during the Zika virus (ZIKV) epidemic on the island of Puerto Rico in 2016. With the demonstration of transfusion transmission and ZIKV infection-associated congenital birth defects, the US Food and Drug Agency (FDA) encouraged Cerus Corporation to make its PR-RBC technology available on the island, first in the form of a Phase III clinical study to demonstrate safety and then, if needed, to the general population. Funded by a contract with the US Department of Health and Human Services, Biomedical Advanced Research and Development Authority (BARDA), the RedeS study sought to enroll 600 general medical and surgical patients for a 28-day transfusion period. Enrollment commenced at three Puerto Rican Hospitals but was interrupted for prolonged periods by hurricanes Irma and Maria in September 2017, followed by earthquakes in 2019 and 2020, and COVID-19 in 2020. Enrollment was closed on the island and following consultation with the FDA, clinical sites were opened on the US mainland and Turkey with expansion of the study to allow a 6-month extension transfusion period to gather data on repeatedly transfused and red cell exchange (RCE) subjects with chronic anemia. The study’s objective is to evaluate the safety and efficacy of INERCEPT RBCs compared to conventional RBCs in patient requiring RBC transfusion support. The primary efficacy endpoint – adjusted post-transfusion Hb increment – will be assessed in a subset of all patients who were non-bleeding at baseline (patients with expected surgical bleeding or RCE were included in the baseline “bleeding” group by design) (Fig. 1). The study is designed to test a non-inferiority hypothesis; namely, that INTERCEPT RBCs are clinically comparable to conventional RBCs in routine transfusion practice. Conventional RBCs were selected as the comparator since they represent the current global standard of care for patients requiring hemoglobin supplementation by transfusion.

Cerus Corporation (Concord, California, USA) is developing the amustaline/GSH pathogen reduction PR system for RBC concentrates to proactively address the risk of transfusion-transmitted infections (TTIs) and transfusion associated graft-versus-host disease (TA-GVHD). The system uses amustaline (S-303), a nucleic acid-targeting molecule, to inactivate infectious pathogens and leukocytes [1]. Amustaline added within 48 h of collection to packed RBCs in additive solution forms irreversible adducts and covalent crosslinks within single or double stranded DNA or RNA to inhibit pathogen and leukocyte replication. GSH is included in the process to reduce unwanted side reactions on the RBC surface [2]. The amustaline/GSH-treatment blocks T-cell replication to replace irradiation as a means of reducing the risk of TA-GVHD [3]. The process includes a terminal media exchange step that reduces the concentration of plasma proteins, including antibodies such that the final product meets the European (EDQM) criteria for washed RBCs [4], which may reduce the incidence of allergic reactions and Transfusion-Related Acute Lung Injury (TRALI). PR-RBCs prepared with the INTERCEPT Blood System for RBCs (hereafter referred to as amustaline/GSH RBCs) may be stored for up to 35 days at 1–6°C before transfusion.

In vitro studies with the amustaline/GSH PR system have demonstrated inactivation of a wide range of bacterial species, enveloped and non-enveloped viruses, protozoa and leukocytes [1, 2, 5]. Amustaline/GSH PR-RBCs have adequate viability in healthy subjects in in vivo recovery and survival studies, [5] and successfully met clinical non-inferiority endpoints in three clinical studies in cardiac surgery and transfusion dependent β-thalassemia patients in Europe and the United States [6–8]. The protocol for a fourth pre-authorization Phase 3 trial in patients requiring transfusion for a broad array of indications, including repeatedly transfused and red cell exchange patients, is presented here.

## Methods/design

CLI 00126 (RedeS) is a randomized, double-blind, actively controlled, parallel group study with 1:1 allocation investigating the INTERCEPT Blood System for red blood cells in regions at potential risk for Zika virus transfusion-transmitted infections. It evaluates the safety and efficacy of amustaline/GSH RBCs compared to conventional RBCs in patients who require acute and long-term RBC transfusion support (clinicaltrials.gov: NCT03037164). The primary safety endpoint is the incidence of related treatment-emergent adverse events (TEAEs) that occur within 28 days of the last study transfusion. Additionally, because treatment-emergent and natural antibodies specific for amustaline/GSH RBCs have been described in patients exposed or never exposed to amustaline/GSH RBCs [9–11], the study is designed to evaluate the incidence, nature and clinical significance [12] of antibodies specific for amustaline/GSH PR-RBCs within 75 days of the last study transfusion.

The primary efficacy endpoint is the post-transfusion adjusted Hb increment assessed in all subjects who were non-bleeding at baseline (subjects with expected surgical bleeding or RCE are defined within the “bleeding” category).

RedeS is sponsored by Cerus Corporation (hereafter referred to as the Sponsor). The trial will be conducted according to the protocol subject to institutional review board (IRB) approval, the International Council on Harmonization E6 Good Clinical Practice (GCP), and applicable local/national regulatory requirements and laws. This report has been formatted using the SPIRIT reporting guidelines,(13) as described in the SPIRIT checklist provided in the supplemental materials. A summary of the study’s schedule of enrollment, interventions, and assessments is provided in Tables 1 and 2.

**Table 1:** Schedule of enrollment, interventions, and assessments: 28-day acute transfusion support and 28-day + 6-month extension period assessments for patients receiving repeated simple transfusions Patient populations: 28-day: Bleeding and non-bleeding patients, all indications; 28-day + 6-Months: Non-bleeding Patients with Thalassemia, SCD, MDS, AA, Chemotherapy or other bone marrow failure syndromes

	**Enrollment & Allocation‡**	**Post-Allocation**			
**Study Assessments**	**Screening**	**Transfusion Support Periods** • **Day 1 through Day 28 *and*** • **28-Day plus 6-month extension (or death-up to 10 transfusion episodes)**		Follow-Up Visit Day 28* (28 ± 7 days) after last study transfusion episode	**End of Study-Day 75 of** ** Follow-Up (75 ± 15 days) after last study transfusion**
		**Before each transfusion episode**	**15 min—24 h. after each transfusion episode**		
Informed Consent (assent, if appropriate)	X^1^				
Medical, surgical, prior transfusion and medication history	X^1^				
Physical exam, including height	X^1^				
Weight	X^1^				
Demographics	X^1^				
ABO Blood type and Rh^3^	X^1^	X^3^			
Indirect Antiglobulin Test^3^	X^1^	X^3^		X	
DAT^8^	X^1^			X	X
Immune reactivity to amustaline/GSH RBCs (amustaline/GSH RBC antibody testing)	X^1^	X^7^		X	X
Pregnancy test (if applicable)^3^	X				
Indication for RBC transfusion	X^1^				
Hematology panel^9^	X^1^	X		X	
Hemoglobin measurement			X^2^		
Blood chemistry panel^10^	X^1^	X		X	
Coagulation panel^11^	X^1^			X	
Vital signs	X^1^	X	X	X	
RBC transfusion data (study or non-study)	X^1^	X		X^5^	
Concomitant medications	X^1^	X^4^		X	
Other blood components transfused		X		X	
Procedure details (as applicable, e.g., surgery)		X			
Adverse events and transfusion reactions		X	X	X^13^	
SAEs		X	X	X^13^	
Surveillance calls ^12^		X		X	
Documentation of vital status		X		X	X
Assess proportion of each RBC component transfused (e.g., 25%, 50%, 75% or 100%) For pediatric patients, the volume of the intended transfusion should be recorded			X		
Assessment of bleeding status^6^	X	X			

‡ Randomization of eligible subjects may occur any time up to 30 days prior to first study transfusion

* Day 1 of the follow-up period is the first day after the last study transfusion

^1^If results from the Screening assessments listed are available in the medical record within 30 days prior to randomization, those data may serve as the screening data and the assessments do not need to be repeated unless the subject has an RBC transfusion in the 30-day period. If an RBC transfusion occurs after determined eligibility but prior to randomization, the subject may be randomized, and those Screening assessments listed above should be repeated prior to the first study transfusion

^2^Hb measurement at 15 min to 24 h post transfusion episode (before the next RBC transfusion episode)

^3^Routine standard of care test order per local hospital SOPs

^4^Medications administered during anesthesia for surgical or other procedures are not collected

^5^The date of non-study RBC transfusion, if any, during the 28-day follow-up period and 6-month extension, if applicable, must be documented

^6^Daily for inpatients when available through the end of the acute transfusion period for inpatients

^7^Performed in conjunction with every routine standard of care test for ABO Type, Rh and screen (IAT) test that is performed during the Acute Support Period

^8^If, during the acute support period or follow up assessments (Day 28), a positive DAT result is observed, participating sites will follow their routine standard practice for confirmation/characterization of DAT positive samples; this should only apply to any conversion from a negative to a positive DAT after enrollment, that is not part of an investigation of hemolysis or of a positive IAT with evidence of an amustaline/GSH RBC antibody, in which case further investigations may be appropriate after discussion with the sponsor

^9^Hematology Panel: WBC, RBC, Hb, Hct, MCV, MCH, MCHC, MPV, RDW, PLT, Neutrophil %, Monocyte %, Lymphocyte %, Basophil %, Eosinophil %

^10^Blood Chemistry Panel: BUN, Creatinine, EGFR, NA, K, GLU, Cl, CO_2_, TP, ALB, ALKP, AST (SGOT), ALT (SGPT), LDH, CPK, TBIL, DBIL

^11^Coagulation Panel: PT, INR, APTT, Fibrinogen

^12^In the outpatient setting if the subject is not seen by study staff for any other reason, surveillance calls are to be completed at least monthly (at minimum every 35 days) to collect AEs, SAEs, TRs, and UADEs starting from the initiation of the first study transfusion through the entire transfusion support period and through 28 days after the final study transfusion. If the monthly surveillance call interval coincides with a study visit, data will be collected at the study visit

**Table 2:** Schedule of Enrollment, Interventions, and Assessments: 28-Day Plus 6-Month Extension for Sickle Cell Disease Patients Undergoing Red Cell Exchange (RCE) Patient population: Sickle Cell Disease patients on a regular, repeated RCE program receiving 3 consecutive RCE procedures. Note: Pre-allocation screening and randomization steps are the same as those in Table 1. The footnote numbering scheme in this table also maps to Table 1

	**Post-Allocation**				
**Study Assessments**	**Transfusion Support Period** **(Day 1 through Day 28 plus 6-month extension or death – 3 RCE episodes)**		**Follow-Up Visit Day 14 (14 ± 7 days) after each RCE episode**	Follow-Up Visit Day 28* (28 ± 7 days) after last study RCE or prior to next non-study RCE if less than 28 days since last study RCE	**End of Study-Day 75 of Follow-Up (75 ± 15 days) after last study transfusion**
	**Before each RCE episode**	**15 min – 4 h. after each RCE episode**			
ABO blood type and Rh^3^. Extended phenotype, if available	X^3^				
Indirect Antiglobulin Test^3^	X^3^			X	
DAT^8^				X	X
Immune reactivity to amustaline/GSH RBCs (amustaline/GSH RBC antibody testing)	X^7^			X	X
Hematology panel and reticulocytes^9^	X	X	X	X	
% HbS, HbA, HbF	X^2^	X^2^	X	X	
Blood chemistry panel^10^	X			X	
Coagulation panel^11^	X	X		X	
S-300 and GSH (frozen plasma)	X	X		X	
Vital signs	X	X		X	
RBC transfusion data (study or non-study)	X			X^5^	
Concomitant medications	X^4^			X	
Other blood components transfused	X			X	
RCE procedure details		X			
Adverse events, AESI and transfusion reactions	X	X	X	X^13^	
SAEs	X	X	X	X^13^	
Surveillance Calls ^12^	X		X	X	
Documentation of vital status	X			X	X
Assess proportion of each RBC component transfused (e.g., 25%, 50%, 75% or 100%) For pediatric patients, the volume of the intended transfusion should be recorded		X			
Assessment of bleeding status^6^	X				

## Participants

Subjects will be recruited in hospital settings in at least 15 healthcare facilities in the US (including Puerto Rico) and Turkey where RBC transfusions are routinely performed for acute and chronic anemia. Site selection criteria for the chronic anemia cohort, including RCE, are dependent on investigators’ access to potential subjects. Clinical research staff at all participating sites will prospectively review patient admissions records, surgical calendars and other hospital records to identify potential subjects. A list of study sites is available on clinicaltrials.gov.

The target population includes patients aged ≥ 4 years who require or are expected to require an RBC transfusion or RCE. Informed consent will be obtained by the site investigators or by authorized and trained study staff using an approved informed consent form (ICF) developed in compliance with ICH GCP, regional regulatory requirements, and applicable laws. Investigators will obtain (and retain in the study file) written informed consent from each patient or the patient's legally acceptable representative before any trial-specific activity is performed. The ICF must be prospectively approved by both the Sponsor and each study site’s IRB or institutional ethics committee (IEC) before use and after any revisions to the protocol. Female subjects of child-bearing potential must have a negative pregnancy test within 30 days of randomization and agree to use at least one method of birth control during the study with a demonstrated low failure rate (< 1%). Patients will be excluded prior to randomization if they meet any of the criteria shown in Table 3. Exclusion criteria apply at the time of randomization. There are no specific exclusion criteria after randomization, with the exception of treatment-emergent antibodies to INTERCEPT RBCs, which would trigger a patient’s withdrawal from the active transfusion component of the trial.

**Table 3:** Exclusion criteria

Patients will be excluded if they meet any of the following criteria prior to randomization: 1. Confirmed positive baseline serum/plasma antibody specific to amustaline/GSH RBCs as determined by amustaline/GSH RBC antibody screening panel prior to receiving the first study transfusion 2. Pregnant or breast feeding 3. Presence of an RBC warm autoantibody with evidence of active hemolysis 4. Positive DAT as defined below: • A polyspecific DAT reaction strength > 2 +, or • A polyspecific DAT (any strength) in conjunction with pan-reactivity with a commercial IAT antibody screening panel that precludes the identification of underlying alloantibodies or indicates the presence of autoantibody 5. Have received investigational products, including investigational blood products, pharmacologic agents or imaging materials, within 28 days prior to randomization. Prior receipt of conventional blood products tested with an investigational NAT test is not considered ground for exclusion 6. Patients presenting with or expected to have massive hemorrhage (≥ 10 RBC units within 24 h) or expected to require massive transfusion protocols. Planned RCE does not apply 7. Patients who require neonatal transfusions and intrauterine transfusions 8. Pre-existing antibody to RBC antigens that may make the provision of compatible study RBC components difficult 9. History of transfusion reactions requiring washed RBCs, volume reduced RBC, or RBCs with additive solution removed 10. Patients with documented IgA deficiency or a history of severe allergic reactions to blood products 11. For SCD patients to be enrolled into the 28-day + 6-month repeated RCE arm of the study: • A history of acute chest syndrome in the last 6 months, or hyperhemolysis syndrome at any time • Clinical evidence of splenic hyperfunction or splenic enlargement: ≥ 18 cm in longitudinal diameter (diagnosed at the Investigator’s discretion according to the data available, with ultrasound data being preferable) • Currently receiving chemotherapy for treatment of cancer. Hydroxyurea for SCD is acceptable if subject has been on stable therapy for 3 months and no changes to dosage are planned • Subject is in active treatment with renal dialysis • Any subject for whom a substantial change in the number of RBC components transfused is anticipated due to anticipated splenectomy, bone marrow transplant, surgery or other change in clinical status • Subject with known G6PD deficiency or requiring treatment with medications that are known to adversely affect RBC viability or bone marrow function	

Patient enrollment for all sites was completed on 01 October 2025. The final patient was randomized on 02 October 2025. Recruitment and enrollment for the study remained open until the last randomized patient was transfused with study RBCs on 24 October 2025. The Sponsor anticipates that the last patient will complete all study assessments in the third quarter of 2026.

### Interventions

The Test device is the INTERCEPT Blood System for RBCs. The INTERCEPT treatment process will be performed on leukocyte-reduced RBC components prepared from whole blood collections from qualified allogeneic blood donors and stored in AS-5 additive solution (or SAG-M at non-US sites). Test PR-RBCs are allogeneic PR-RBCs stored at 1 °C to 6 °C in SAG-M additive solution for up to 35 days post-donation and administered intravenously. Test products may be irradiated if indicated by the treating physician. Dose and schedule of RBC transfusions will be determined by the treating physician. Control RBCs are conventional leukocyte-reduced RBCs stored in AS-1 or AS-5 additive solution RBCs stored at 1 °C to 6 °C for up to 35 days post-donation.

Study RBCs (both Test and Control) will be labeled as investigational products in a manner that protects the study blind and may not be used in routine care of non-study patients.

### Treatment plan

**Fig. 2:**
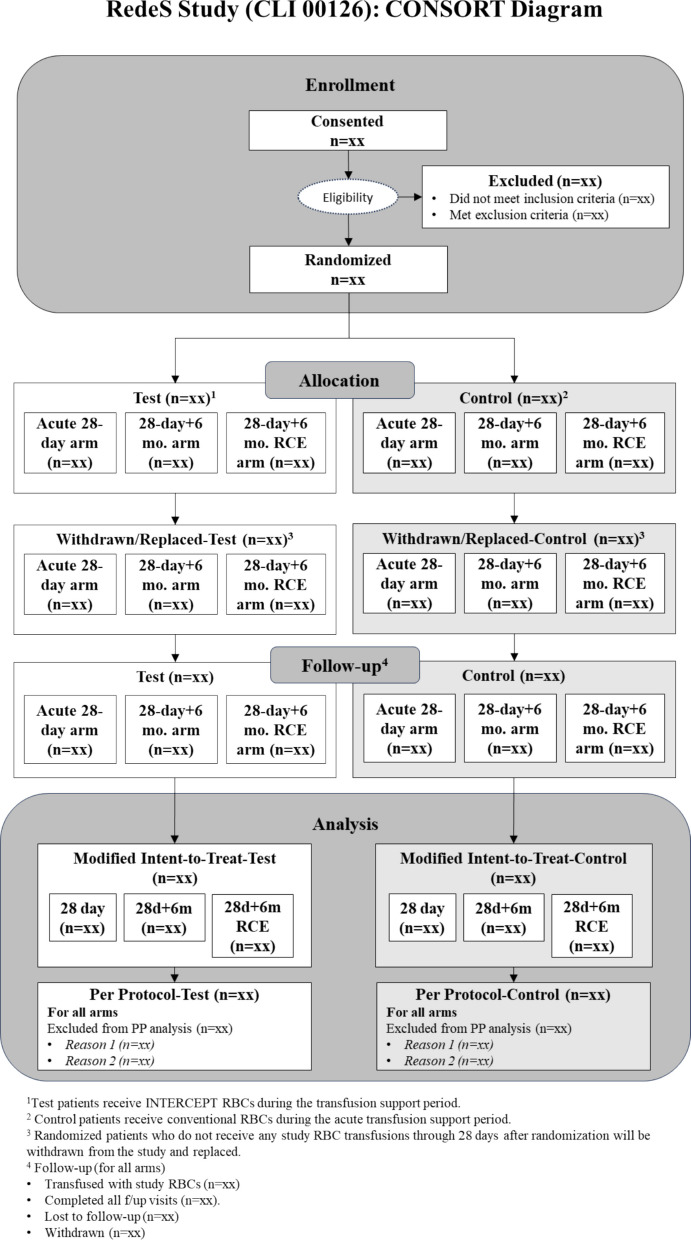
Consort Diagram of Study Flow

**Fig. 3:**
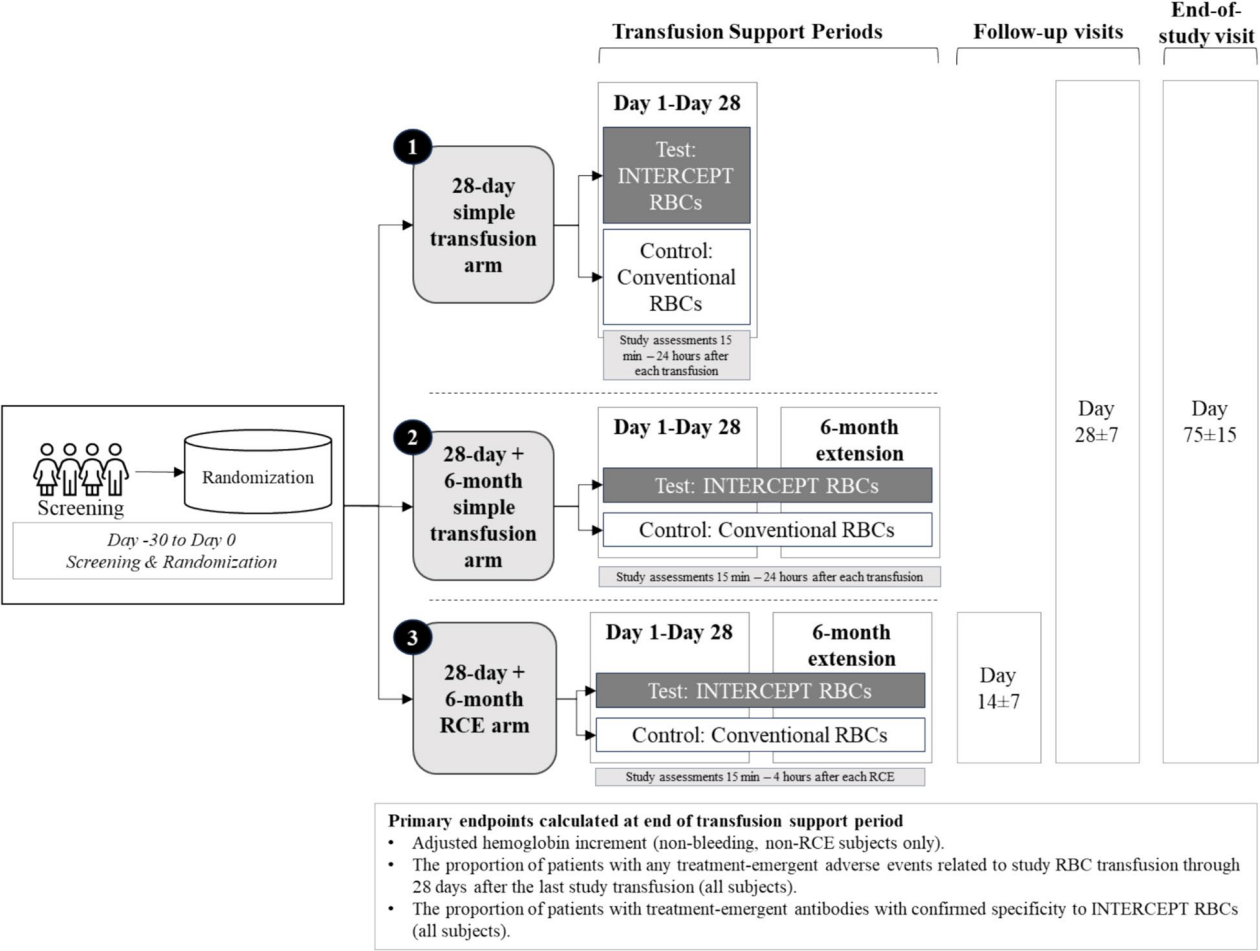
Study enrollment flowchart and assessment timeline

The study’s enrollment plan is shown in the CONSORT diagram in Fig. 2. The study assignment flow chart and assessment timeline is shown in Fig. 3. The schedule of assessments is shown in Tables 1 and 2.

### Screening/randomization (day −30 to day 0 pre-surgery)

Patients will be approached, consented (or assented, if appropriate), screened for eligibility, and randomized into one of the study arms (28 day acute; 28-day + 6-month extension) by investigators at each site. Subjects < 18 years of age will require subject assent and parental consent. For subjects eligible for enrollment into the six-month extension period, consent/assent for the extension period will be obtained at initial enrollment. Patients who consent to the study will be assigned a study ID number and undergo screening procedures described in Tables 1 and 2.

Screening data collection and procedures will include: demographics (age, sex), vital signs, height, weight, indication for anticipated transfusion, type of scheduled surgery (if applicable), medical and surgical history, transfusion history, physical examination, comorbid conditions, concomitant medications, hematology panel, blood type and Rhesus factor, indirect antibody test (IAT), blood chemistry panel, coagulation panel, direct antibody test (DAT), immune reactivity to amustaline/GSH RBCs, and pregnancy test (when applicable; performed within 30 days of randomization). The assays included in each of the panels referenced above are listed in the footnotes of Tables 1 and 2.

If results from the Screening assessments listed in Table 1 are available in the medical record within 30 days prior to randomization, those data may serve as the screening data and the assessments do not need to be repeated unless the subject has a non-study RBC transfusion in the 30-day period. If a non-study RBC transfusion occurs after a subject is determined to be eligible, but prior to randomization, the subject may be randomized and screening assessments will be repeated prior to the first study transfusion.

For SCD patients on a regular RCE program the pre- and post- RCE total Hb, HbA, HbS and HbF concentrations should be recorded from the medical record, if available, for up to 6 months prior to the first study transfusion (Table 2).

### Randomization

An Interactive Web Response System (IWRS) will be used for electronic randomization of eligible patients. At the end of screening and prior to the first study RBC transfusion, the patient’s eligibility status will be entered into the study EDC system. If eligible, the patient will be randomized to receive either amustaline/GSH or conventional RBCs in a 1:1 ratio. Randomization will be stratified by site, baseline bleeding status (indication for RBC transfusion due to active bleeding or for anemia without active bleeding), patients’ participation in the optional extension period, and patients’ need for RCE (Fig. 2). Active bleeding will be defined as Grade 3 or 4 bleeding of the expanded WHO bleeding scale. Anticipated surgical bleeding and planned blood loss/replacement through RCE at baseline should be included as active bleeding (Grade ≥ 3) by definition.

### Transfusion support periods: 28-day acute transfusion support or 28-day + 6-month extension

Subjects will receive amustaline/GSH (Test) or conventional (Control) RBCs during the transfusion support period into which they have been enrolled, or until death, whichever is first. The day of the first study transfusion will be considered study Day 1 for defining the transfusion support period. Transfusions will be administered according to local institutional policy and safety standards as ordered by the medical team.

Data on all AEs, serious AEs (SAEs), transfusion reactions (TRs) and/or Unanticipated Adverse Device Effects (UADEs) will be collected from the initiation of the first study RBC transfusion through 28 days after the last study RBC transfusion. SAEs are required to be reported by the Investigator to the Sponsor or designee immediately (i.e., no more than 24 h after learning of the event). In an inpatient setting bleeding status and safety events will be collected daily following each study RBC transfusion until discharge or death, whichever is first. In the outpatient setting surveillance calls are to be completed at least monthly (at minimum every 35 days) if the subject is not seen by study staff for any other reason. If the monthly surveillance call interval coincides with a study visit, these data may be collected as part of the study visit assessments described in Tables 1 and 2.

For pediatric patients, the volume of the intended transfusion should be recorded. Study staff will record the proportion of the transfused unit. If for any reason a unit is not fully transfused, the percentage of the bag that was transfused (i.e., 75%, 50%, 25%) and the reason the remainder of the unit was not transfused or the why the transfusion was stopped will be recorded. This proportion will be recorded for each unit of study RBCs transfused and combined with the Hb concentration and net volume of the RBC component as measured by the blood center. These variables will be used to calculate the actual Hb mass transfused.

A negative antibody screen for amustaline/GSH RBCs should be confirmed prior to transfusing a patient with study RBCs and each time a standard of care ABO Type, Rh and screen (IAT) test is performed within the 28-day acute transfusion period and the 6-month extension period, if applicable.

The 28-day + 6-month extension period will include a subset of SCD patients who will receive three consecutive RCEs with study RBCs. For SCD patients undergoing regular RCE, the parameters listed in Table 2 should be assessed before and 15 min to 4 h after each RCE procedure. These assessments will be performed at 14-day (± 7 days) intervals between each of the 3 study RCE episodes.

Study RBC components will be ordered and administered to study patients by their treating physicians according to local standards of care. Randomized patients who do not receive any RBC transfusion within 28 days of randomization will be withdrawn and replaced. No additional data will be collected for these patients, who will not be included in the data analysis. These subjects may be reassessed for eligibility and re-enrollment after withdrawal.

If a subject is determined to have developed a treatment-emergent antibody to amustaline/GSH RBCs, the subject should be withdrawn from the active treatment phase of the study but should continue with all planned safety assessments per protocol. If the subject requires further transfusion, conventional RBC should be transfused. The subject should be actively investigated for signs or symptoms of intravascular or extravascular hemolysis for at least 4 weeks after the last study transfusion. Blood samples should be sent to the study’s central immunohematology reference center (Versiti Blood Center of Wisconsin, formerly Blood Center of Wisconsin) for assessment of the hemolytic potential and changing characteristics and titer of amustaline/GSH antibodies every 1–2 weeks for 4 weeks or until resolution of the event.

### 28-day (± 7 days) post-transfusion support follow-up period

Day 1 of the 28-day (± 7 days) follow-up period is the first day after the last study transfusion. The last study transfusion will occur no later than the end of the 28-day acute transfusion period or the 6-month extension period. For subjects enrolled in the 6-month extension period, the last study transfusion may also be the tenth transfusion episode for repeated simple transfusion subjects, or the third RCE episode in SCD subjects. At least one monthly surveillance call will be performed with all subjects to collect data on AEs, SAEs, TRs and UADEs during the 28-day (± 7 days) follow-up period. On Day 28 (± *7* days) after the last study transfusion, subjects will return to the study site for the following assessments: Vital signs, AEs, SAEs, TRs, comorbid conditions, blood samples for safety labs (hematology, blood chemistry, and coagulation panels), IAT and DAT, and immune reactivity to amustaline/GSH RBCs. Samples may be used for additional in vitro research testing as required to confirm transfusion-transmitted pathogen mediated infections.

For SCD patients undergoing regular RCE, the assessments described in Table 4 should be collected on Day 28 (± *7* days).

**Table 4:** Laboratory Assessments for SCD Subjects Enrolled in the RCE Arm

• Hematology panel• Reticulocytes • Proportion (%) of HbS, HbA, and HbF • Coagulation panel • Plasma frozen sample for determination of S-300 and GSH concentrations	

### Day 75 (± 15 days) end-of-study visit

On Day 75 (± 15 days) of the follow-up period after the last study RBC transfusion, a blood sample will be collected for a DAT and an immune reactivity test for antibodies specific for PR-RBCs.

### Patient withdrawal criteria

Study patients are free to withdraw consent and discontinue/withdraw from the study at any time without prejudice to further treatment. A patient’s participation in the study may be withdrawn at any time at the discretion of the Investigator if he/she feels it is in the patient’s best interest.

Upon early termination or withdrawal of patients who have received at least one study RBC transfusion, the tests required during the Day 28 (± 7 days) and/or Day 75 (± 15 days) visits will be performed. If this is not possible then, depending upon the study period in which the patient was withdrawn, a physical examination and standard laboratory tests should be performed; other blood samples should be collected (as needed); and data on concomitant medications and AEs should be obtained.

### Enrollment pauses and stopping rules

The study may be temporarily paused or definitively stopped based on poor or slow subject accrual or for safety recommendations from the Data Safety Monitoring Board (DSMB).

A delayed serologic transfusion reaction (DSTR), delayed hemolytic transfusion reaction (DHTR), acute hemolytic transfusion reaction (AHTR), or hyperhemolysis syndrome in subjects exposed to study RBCs may result in individual patient withdrawal, enrollment pause, or a clinical stop. All subject recruitment, enrollment, randomization, transfusion and clinical follow-up activities will stop during a “clinical stop.” In certain cases, enrollment or transfusion of study patients may be resumed after a stop with the authorization of the DSMB and/or FDA. Subjects will continue to receive non-study conventional RBCs during a clinical stop, as needed. All safety monitoring activities will continue per protocol during a clinical stop.

### Sample size

The study was originally designed to enroll ~ 600 subjects to allow 95% probability that any AEs that occurs at a 1% rate would be documented in subjects that are bleeding or non-bleeding at baseline. With the later amendments to include patients undergoing chronic transfusions including RCE, the total enrollment target was increased to 600–800 enrolled subjects for the primary safety endpoints. The primary safety endpoint will be assessed in the modified intention-to-treat (mITT) population that receive at least one study RBC transfusion, a subset of the enrolled subjects. The primary efficacy endpoint will be assessed in the subset of the mITT that was non-bleeding at baseline, as active bleeding confounds the assessment of Hb increment. At least 352 non-bleeding evaluable patients (approximately 174 per treatment group) will allow for 80% power to declare non-inferiority for the primary efficacy endpoint treatment difference with a non-inferiority margin of 15% of the control mean at the two-sided 0.05 alpha level, assuming no true treatment difference and a coefficient of variance (CV) of 50%. Under the same assumptions, 468 non-bleeding evaluable subjects (234 per treatment group) will provide 90% power.

The study will also aim to enroll one-hundred-and-forty 28-day + 6-month extension subjects, including up to 30 SCD patients requiring regular repeated RCE to allow a total of three consecutive RCE procedures in at least 20 transfused evaluable patients to provide experience with repeatedly transfused subjects. Between 600 to 800 randomized subjects will be enrolled to meet the sample size targets to power the primary efficacy endpoint and the 28-day + 6-month extension period enrollment goals.

### Blinding

Clinical staff and others caring for participating patients, as well as the Sponsor (and delegates), will be blinded to treatment assignment. Unblinded delegates will monitor the production of study RBC components at designated blood centers and distribution at hospital transfusion centers. Only appropriate blood bank and transfusion service staff will be able to access the treatment arm assignment. Study RBC component labels will be indistinguishable for Test and Control products. In order to protect the blind, the final products (Test and Control) will be transferred into an FDA approved RBC storage container (Fenwal™ Transfer Pack™ Container – 600 mL, product code 4R2023) using a sterile docking technique. This transfer will occur on the day of each planned study transfusion.

### Data management

Patients’ medical records are considered the source data. Study data will be recorded on electronic case report forms (eCRF) stored in a 21 CFR Part 11-compliant electronic data capture (EDC) system selected by the Sponsor and maintained by a third-party vendor (InForm, Oracle, Austin, TX, USA). Batch production records and data reports from the blood centers’ electronic data management systems may be considered source data for processing study RBC components. Source data will be verified against the EDC system by the Sponsor and/or by a contract research organization (CRO) selected by the Sponsor (Precision for Medicine, Carlsbad, CA, USA). All study documents will be entered into an electronic trial master file (TMF) maintained by the Sponsor.

### Confidentiality

Individual patient medical information obtained as a result of this study is considered confidential, and disclosure to third parties other than those described below is prohibited. Data generated as a result of this study are to be available for inspection upon request by local health authority auditors, the Sponsor’s monitors, or by a participating site’s IRB/IEC.

### Study coordination and management

Routine study management (including oversight of external partners and contract research organizations, as needed) will be conducted by the Sponsor through a team of Clinical Research Associates and Medical Monitors led by a Clinical Trial Manager and supervised by the head of Clinical Research and the Chief Medical Officer. The study team will hold routine (often daily) interactions with study sites and external partners, as needed, as well as regularly scheduled weekly, monthly and quarterly study review meetings.

## Outcomes

### Primary and secondary endpoints

The primary safety endpoints include the proportion of subjects with any TEAEs possibly, probably or definitely related to a study RBC through 28-days after the last study transfusion, and the proportion of subjects with treatment-emergent antibodies with confirmed specificity to PR-RBCs. The primary efficacy endpoint is the adjusted Hb increment averaged over multiple transfusions for all non-bleeding subjects at baseline, defined as the difference between pre- and 15-min-24 h post-transfusion Hb values divided by the total Hb content transfused during each transfusion episode.

Secondary efficacy and safety endpoints will include:

- Adjusted Hb consumption defined as total Hb mass transfused (g) divided by body weight (kg) and the duration of the study transfusion period (days) in patients without active bleeding at baseline.
- HbA clearance in SCD patients undergoing regular RCE.
- All TEAEs for 28 days after last study transfusion.
- Transfusion reactions related to study RBCs (as defined by the CDC National Healthcare Safety Network [NHSN] Hemovigilance Module protocol), for 28 days after the last study transfusion.
- Treatment-emergent immunization to RBC alloantigens.
- All-cause mortality through 28 days after the last study transfusion.

Secondary Safety Endpoints (for SCD patients undergoing RCE):

- Proportion of subjects with adverse events of special interest (AESI) through 28 days after the last study transfusion.
- S-300 and GSH plasma levels before and 15 min to 4 h after RCE.

### Statistical methods

Only patients in the mITT analysis set will be included in the efficacy analysis. Non-study RBCs received during the study transfusion period will be included to calculate the total Hb content transfused for the two efficacy endpoints in the mITT analysis set. Patients receiving non-study RBC transfusions as their first study transfusion will be excluded from the Per Protocol (PP) analysis set and only those transfusions that occur before a non-study transfusion will be analyzed. Data from the initial 28-day period and the optional 6-month extension period will be summarized together and separately.

Treatment difference in non-bleeding mITT patients will be assessed using a non-inferiority test with a pre-specified non-inferiority margin (15% of the Control mean value), stratified by site. An analysis of variance (ANOVA) model will be used to estimate and test the treatment effects while controlling for the clinical site. Non-inferiority will be declared if the lower bound of a 2-sided 95% confidence interval for the mean treatment difference (Test—0.85 × Control) is greater than or equal to zero.

For the primary efficacy endpoint, the adjusted Hb increment will be averaged over one or more transfusion episodes. An episode-specific Hb increment will be calculated by subtracting the Hb value before each transfusion episode from the Hb value taken 15-min-to-24-h after each transfusion episode. The relatively long post-transfusion assessment period is justified by prior findings that post-transfusion hemoglobin increments are not meaningfully impacted by the timing of the samples collected between 15 min and 24-h post-transfusion [14, 15]. Increments will be adjusted by the Hb content transfused in each given episode (which may contain multiple RBC components). The Hb content of each transfused RBC component will be calculated using the Hb concentration and post-sample weight of the RBC component (both measured at the blood center), and the percentage of the unit transfused (assessed at the hospital). The Hb mass of each transfusion episode is the sum of the Hb content for all RBC components transfused.

The Hb content transfused (g) for a study RBC component will be calculated as:

$$post sampling weight \left(g\right)\div 1.06 \left(\frac{g}{mL}\right)\times percent of unit transfused \times hemoglobin concentration (g/mL)\div 100$$

Non-study RBCs received during the study transfusion period will be included in the calculation of Hb content transfused. For each non-study RBC component received, the Hb content will be imputed using the average Hb content per study RBC component from the Control group within each clinical site.

The treatment difference in the adjusted Hb increment will also be summarized descriptively (with a 95% confidence interval) as exploratory analyses for the following mITT subgroups:

- Repeatedly transfused non-bleeding patients (defined as non-bleeding patients who receive at least 3 study RBC episodes).
- Subgroup by clinical site, age group (< 18, 18–64, and ≥ 65 years old), sex, and race (e.g., white vs. other races), as appropriate.

For SCD patients enrolled in the 6-month extension for three RCE procedures, HbA clearance will be estimated from the area under the curve (AUC) calculated by the trapezoidal rule based on measurements from samples collected within 4-h of each RCE, on Day 14 (± 7-days) and prior to the next scheduled RCE. Values will be adjusted by the duration in days used to calculate the AUC (i.e., between the 15 min-to-4-h post-transfusion sample and pre-transfusion sample of the next transfusion) and averaged over all study RCE procedures for each patient.

For the secondary efficacy endpoint of Hb consumption, the analysis will be conducted only in non-bleeding mITT patients who participate in both of the initial 28-day and 6-month extension periods and will utilize integrated data from both periods. Treatment differences in non-bleeding mITT patients will be assessed using the same non-inferiority test methods described above for the primary endpoint analysis. Interaction terms between the treatment and covariates will be explored, as necessary. For the adjusted Hb consumption analysis, the upper bound of a two-sided 95% confidence interval for the treatment difference (Test – 1.15 × Control) less than or equal to zero will result in the rejection of inferiority null hypothesis and the conclusion of non-inferiority.

Adjusted Hb consumption will be calculated as total Hb mass transfused in grams divided by body weight in kg at baseline and duration of the study transfusion period in days (g/kg/day). The total Hb mass transfused is the sum of the Hb content for all study and non-study RBC components transfused during the study transfusion period (i.e., 28 days or 28-day + 6-months, unless subjects prematurely discontinue the study).

For patients who prematurely discontinue from the study, the duration of the study transfusion period will be calculated as:

$$\left(end\;date\;of\;study\;transfusion\;period\right)-\left(start\;date\;of\;first\;study\;RBC\;component\right){+1}$$

Where the end date of the study transfusion period will be defined according to the following rules:

- For subjects who withdraw due to death, the date of death will be the end date of the study transfusion period.
- For patients withdrawn for reasons other than death (e.g., lost to follow-up, withdrawal of consent, AE), the last date of a study or non-study RBC transfusion during the planned study transfusion period (28-days or 28-day + 6-months) will be the end date of the study transfusion period.

The treatment difference in the adjusted Hb consumption will also be summarized descriptively as exploratory analyses for the following mITT subgroups:

- Repeatedly transfused non-bleeding patients (defined as non-bleeding patients who receive at least 3 study RBC episodes).
- Subgroup by clinical site, age group (< 18, 18–64, and ≥ 65 years old), sex, and race, as appropriate.

Only mITT patients will be included in the safety analysis. Treatment-emergent AEs are defined as AEs with an onset date/time that is on or after the start date/time of the first study RBC transfusion. AEs will be summarized by treatment group, system organ class (SOC), and preferred term as applicable. The proportion of patients with AEs possibly, probably, or definitely related to study RBC transfusion will be compared between treatment groups using a Cochran-Mantel–Haenszel (CMH) test (controlling for clinical site and baseline bleeding status). The risk difference associated confidence interval will also be reported. In the event of sparse counts, Fisher’s exact test may also be used to provide statistical interpretation.

To investigate the potential impact of irradiation, subgroup analysis by irradiation status (yes vs. no) may be presented for the primary efficacy endpoint and selected safety data (including SAEs, AEs, and potassium level).

### Interim review

After approximately 300 patients are treated and at any other time that safety or enrollment rate concerns arise, safety data will be summarized in a group blinded manner for the DSMB's interim safety data review. Enrollment and study transfusions will continue during the interim safety evaluation, unless otherwise advised by the DSMB. Depending on the group blinded safety data reviewed, the DSMB may call for an additional unscheduled review, request to see unblinding summary, and/or suggest holding, stopping, or modifying the study for safety concerns.

As this planned interim analysis can only stop the trial for safety concerns, no adjustments will be made for the alpha level. If the study is discontinued as a result of this interim safety review, unblinded safety and efficacy data will be analyzed then and submitted to the DSMB and FDA.

### Data and Safety Monitoring Board (DSMB)

Study data will be monitored on a regular basis by a DSMB that is independent of the Sponsor, funder (BARDA) and Investigators. The primary responsibility of the DSMB is to ensure patient safety. DSMB members will be independent of the Sponsor. The Sponsor will notify the DSMB of fatal outcomes assessed to be possibly, probably, or definitely related to a study RBC and any confirmed amustaline/GSH RBC antibodies within 72 h of becoming aware of the event. DSMB members will be selected based on relevant academic and real-world training and experience. The DSMB will include specialists in blood banking, immunology, biostatistics and clinical medicine. The work of the DSMB will be guided by a charter developed by the Sponsor and agreed by all members of the board.

## Discussion

Over the last decade epidemics of multiple vector-borne arboviruses (e.g., Dengue [DENV], Chikungunya [CHIKV] and Zika [ZIKV] viruses) with the potential for transmission via transfusion have emerged in several areas of the world [16]. ZIKV, in particular, has major implications for blood safety and availability [17] as it may be transmitted by transfusion [18–20] and has potential deleterious outcomes such as microcephaly [21, 22] and Guillain–Barre syndrome [23]. In 2016, FDA issued guidance recommending the cessation of blood collections in areas with active community transmission of ZIKV, such as Puerto Rico. Platelets and plasma could be collected if an approved pathogen reduction device was used [24]. The US Government response to the 2014–2016 ZIKV outbreak in the Americas also included funding for the amustaline/GSH PR-RBC study described here. Subsequent arboviral outbreaks and the COVID-19 pandemic have led to increasing interest in pathogen reduction technologies (PRT) for RBCs as an additional safety measure for blood components [25–31].

Pathogen reduction strategies are approved in the US for platelet and plasma components. Current strategies to reduce the risk of TTI for RBC components include only pre-donation evaluation and selection of low-risk donors followed by serologic and/or nucleic acid testing (NAT) for selected known infectious pathogens. Limits to the level of sensitivity achievable with current testing technologies and algorithms and emerging pathogens that may contaminate the blood supply for a period of time prior to discovery remain a concern [32, 33]. Approved tests are not available for all of the infectious agents known to contaminate blood components, and while bacterial culture screening systems are widely used for platelets (which can be derived from whole blood donations that also produce RBC and plasma components), RBC components are not routinely screened for bacterial contamination [32, 34–37]. Unlike infectious risks, TA-GVHD risk can be effectively mitigated by irradiation. However, approximately half of TA-GVHD cases occur in patients who do not qualify for irradiated blood products under current guidelines in Europe and North America, leading to substantial morbidity and mortality among impacted patients [38].

In this setting, the RedeS study aims to demonstrate the safety of amustaline/GSH PR-RBCs in subjects requiring acute and chronic transfusion support, including SCD patients undergoing RCE therapy. The ZIKV outbreak presented a unique opportunity to test the technology in an emergency use scenario in Puerto Rico where the virus was circulating in blood donors [29, 39, 40]. The waning of the ZIKV outbreak led to RedeS protocol amendments associated with broader subject enrollment strategies, notably the expansion to hospitals on the US mainland and Turkey, and the 6-month extension for subjects requiring chronic RBC transfusion support or for SCD patients undergoing repeated RCE procedures.

The primary endpoint, Hb increment, is the most commonly measured indirect indicator of response to transfusion and is widely used clinically to guide transfusion therapy decisions and assess transfusion response. Currently there are no validated outcome measures of RBC efficacy that assesses tissue oxygenation directly. With bleeding patients, the interpretation of post-transfusion Hb increment may be complicated by Hb loss due to bleeding and the dilutional effects of administering non-RBC-containing intravenous fluids. For these reasons, the study endpoints will be evaluated separately within the bleeding and non-bleeding subject populations.

An important secondary endpoint of the study is the incidence and clinical significance of antibodies to neoantigens formed as a result of amustaline and/or GSH binding to RBC surface membrane proteins or lipids during the pathogen reduction process. Naturally occurring antibodies with specificity to these RBCs have also been detected in surveillance studies of blood donors and patients transfused with conventional RBCs [10]. Treatment emergent antibodies specific to amustaline/GSH treated RBCs were observed with an earlier version of the amustaline/GSH pathogen reduction device, which was reconfigured to include a higher dose of GSH to further quench RBC side-reactions. The reconfigured amustaline/GSH device showed reduced immunogenicity in animal models and did not result in treatment-emergent antibodies in two randomized clinical trials in cardiac surgery patients and in transfusion-dependent β-thalassemia patients in Europe and Turkey [2, 6, 7]. In the United States-based ReCePI trial, [41] five of 159 test subjects (3.1%) developed treatment-emergent antibodies with specificity to amustaline/GSH RBCs. Most of these treatment-emergent and naturally occurring antibodies were neutralized by soluble acridine, suggesting specificity to acridine-containing adducts; [10] however, some antibodies have been detected that were not inhibited by acridine. To date, none of these antibodies have resulted in definite signs or symptoms of hemolysis and there have been no signs of decreased RBC lifespan. The five antibodies detected in the ReCePI study were detected 26–80 days post-surgery. Four of the five antibodies had negative DAT results (one was weakly positive with an amustaline/GSH-specific eluate). A monocyte monolayer RBC phagocytic assay (MMA) performed on fresh amustaline/GSH RBCs with high acridine surface density was non-reactive for three of the 5 subjects. Subjects had a mean exposure of 1–3 amustaline/GSH RBCs prior to developing antibodies. Flow cytometry tuned to detect acridine on RBC surface membranes was able to track persistent circulating amustaline/GSH-treated RBCs, indicating a lack of accelerated clearance [11].

The RedeS study is designed to exclude subjects with natural antibodies to amustaline/GSH RBCs at baseline and to detect and investigate the clinical significance of treatment emergent antibodies. The occurrence of a single subject with evidence of RBC hemolysis associated with an amustaline/GSH antibody would require a clinical hold with evaluation and DSMB and FDA approval to continue enrollment. No hemolytic antibodies have been reported at the time of this publication and none have been observed in any previous or on-going clinical study with amustaline/GSH RBCs. A small number (n = 4) of non-clinically significant, low titer antibodies reactive to the amustaline/GSH screening panel have been detected in the RedeS study to date. The study remains blinded and it is not known whether those subjects received Test or Control study RBCs. Documenting the rate and nature of (e.g., titer, immunoglobulin isotype) the humoral immune response to transfused RBCs as well as any potential clinical consequences (e.g., increased RBC clearance) will be critical to interpreting and understanding the potential clinical significance of these antibodies.

Other secondary efficacy endpoints include adjusted Hb consumption, the duration of study transfusion periods (days) in patients without active bleeding at baseline, and HbA clearance in SCD patients undergoing regular RCE. While comparable Hb consumption and transfusion support periods have been observed in prior clinical studies with amustaline/GSH RBCs, [7] little is known about Hb clearance in SCD patients on chronic simple transfusion therapy with amustaline/GSH RBCs compared to conventional RBCs (42). Likewise, HbA clearance in SCD patients receiving RCE therapy with amustaline/GSH RBCs has not been described previously.

This study design is associated with a number of potential limitations. In the absence of an acute epidemic or pandemic threat, enrollment and subject selection in transfusion medicine studies can be challenging due to competing trials and lack of patient awareness of transfusion medicine topics. Furthermore, the 6-month extension of this study has a significant on-study time commitment and potentially high number of study related activities (depending on the number of transfusions occurring during that period). Amendment 9 of the protocol limits the number of on-study transfusions to a maximum of ten. This change is designed to streamline recruitment in the 6-month extension arm and improve representativeness of the long-term cohorts as much as possible.

## Trial status

RedeS initially opened for enrollment at three clinical sites in Puerto Rico and transfused the first subject on 03 April 2017. Since then, the Puerto Rican sites have closed to enrollment and an additional 14 sites have been initiated on the US mainland, and at a single site in Turkey. The current protocol (CLI 00126 version 9.0) was revised to reflect the addition of the 6-month extension period and the inclusion of SCD patients undergoing RCE. The current protocol also reflects an adjustment in the study’s enrollment target(s) from 600 to 600–800 subjects, and updates to the statistical analysis plan, which is provided in the supplemental materials. Enrollment was severely impacted by the COVID-19 pandemic between 2020 and 2022, and was successfully closed on 24 October 2025.

## Supplementary Information

Supplementary Material 1.

## Data Availability

Consent forms and trial data will be available upon request from the principal investigator. The Protocol and statistical analysis plan will be published along with the clinical outcomes of the study on clinicaltrials.gov (https://clinicaltrials.gov/study/NCT03037164).

## Abbreviations

AE: Adverse event
AHTR: Acute hemolytic transfusion reaction
BARDA: Biological Advanced Research and Development Authority
CHIKV: Chikungunya virus
CPD: Citrate phosphate dextrose
CTCAE: Common Terminology Criteria for Adverse Event
DAT: Direct Antiglobulin Test (also referred to as the direct Coombs test)
DHTR: Delayed hemolytic transfusion reaction
DSMB: Data Safety Monitoring Board
DSTR: Delayed serological transfusion teaction
FNHTR: Febrile non-hemolytic transfusion reaction
G6PD: Glucose 6-phosphate dehydrogenase
GSH: Glutathione (γ-glutamylcysteinyl-glycine)
Hb: Hemoglobin
HBV: Hepatitis B virus
Hct: Hematocrit
HCV: Hepatitis C virus
HIV: Human immunodeficiency virus
HTR: Hemolytic transfusion reaction
IAT: Indirect Antiglobulin Test (also referred to as the Indirect Coombs test)
mITT: Modified intent-to-treat
PP: Per protocol
RBC: Red blood cell
RCE: Red cell exchange
S-300: (N-(9-Acridinyl)-ß-alanine) (the primary decomposition product of S 303)
S-303: [N,N-bis (2-chloroethyl)]-2-aminoethyl 3-[(acridin-9-yl) amino] propionate (also referred to as amustaline, the primary pathogen inactivation agent in the INTERCEPT Blood System for RBCs)
SAG-M: SAG-Mannitol (an RBC additive solution)
SAE: Serious Adverse Event
SCD: Sickle Cell Disease
TA-GVHD: Transfusion Associated Graft versus Host Disease
TEAE: Treatment-Emergent Adverse Event
TR: Transfusion Reaction
TRALI: Transfusion Related Acute Lung Injury
TTI: Transfusion-Transmitted Infection
WNV: West Nile Virus
ZIKV: Zika Virus

## References

[1] Mufti NA, Erickson AC, North AK, Hanson D, Sawyer L, Corash LM, et al. Treatment of whole blood (WB) and red blood cells (RBC) with S-303 inactivates pathogens and retains in vitro quality of stored RBC. Biologicals. 2010;38(1):14–9. 19995680 10.1016/j.biologicals.2009.10.019

[2] Henschler R, Seifried E, Mufti N. Development of the S-303 pathogen inactivation technology for red blood cell concentrates. Transfus Med Hemother. 2011;38(1):33–42. 21779204 10.1159/000324458 PMC3132978

[3] Castro G, Stassinopoulos A. Effective inactivation of T-cells with amustaline/GSH in human RBC as assessed by fluorescent limiting dilution assays (LDA). Transfus Clin Biol. 2016;23(N°4):308.

[4] European Directorate for the Quality of Medicines and Healthcare (EDQM). Guide to the preparation, use and quality assurance of blood components. 21st Edition. Strasbourg, France: EDQM; 2023.

[5] Cancelas JA, Gottschall JL, Rugg N, Graminske S, Schott MA, North A, et al. Red blood cell concentrates treated with the amustaline (S-303) pathogen reduction system and stored for 35 days retain post-transfusion viability: results of a two-centre study. Vox Sang. 2017;112(3):210–8. 28220519 10.1111/vox.12500

[6] Brixner V, Kiessling AH, Madlener K, Muller MM, Leibacher J, Dombos S, et al. Red blood cells treated with the amustaline (S-303) pathogen reduction system: a transfusion study in cardiac surgery. Transfusion. 2018;58(4):905–16. 29498049 10.1111/trf.14528

[7] Aydinok Y, Piga A, Origa R, Mufti N, Erickson A, North A, et al. Amustaline-glutathione pathogen-reduced red blood cell concentrates for transfusion-dependent thalassaemia. Br J Haematol. 2019;186(4):625–36. 31148155 10.1111/bjh.15963 PMC6771954

[8] Sekela ME, Snyder E, Welsby IJ, Toyoda Y, Sodha NR, Beaver TM, et al. Acute kidney injury to evaluate amustaline/glutathione pathogen reduced red cells in cardiac surgery: outcomes of the ReCePI Phase III Clinical Trial (AABB Annual Meeting oral abstract PL1-SN-25). Transfusion. 2024;64(S3):9A.

[9] Benjamin RJ, McCullough J, Mintz PD, Snyder E, Spotnitz WD, Rizzo RJ, et al. Therapeutic efficacy and safety of red blood cells treated with a chemical process (S-303) for pathogen inactivation: a Phase III clinical trial in cardiac surgery patients. Transfusion. 2005;45(11):1739–49. 16271099 10.1111/j.1537-2995.2005.00583.x

[10] Geisen C, North A, Becker L, Brixner V, von Goetz M, Corash L, et al. Prevalence of natural and acquired antibodies to amustaline/glutathione pathogen reduced red blood cells. Transfusion. 2020;60(10):2389–98. 32692456 10.1111/trf.15965

[11] Karim C, Panigrahi A, Pearl RG, Sodha NR, Beaver TM, Pelletier JPR, et al. Characterizing the antibody response to amustaline/glutathione pathogen-reduced red blood cells. Transfusion. 2025;65(2):344–53. 39719927 10.1111/trf.18117 PMC11826292

[12] Garratty G. What is a clinically significant antibody? ISBT Sci Ser. 2012;7(1):54–7.

[13] Chan AW, Tetzlaff JM, Gøtzsche PC, Altman DG, Mann H, Berlin JA, et al. SPIRIT 2013 explanation and elaboration: guidance for protocols of clinical trials. BMJ. 2013;346:e7586. 23303884 10.1136/bmj.e7586 PMC3541470

[14] Roubinian NH, Plimier C, Woo JP, Lee C, Bruhn R, Liu VX, et al. Effect of donor, component, and recipient characteristics on hemoglobin increments following red blood cell transfusion. Blood. 2019;134(13):1003–13. 31350268 10.1182/blood.2019000773 PMC6764268

[15] Elizalde JI, Clemente J, Marín JL, Panés J, Aragón B, Mas A, et al. Early changes in hemoglobin and hematocrit levels after packed red cell transfusion in patients with acute anemia. Transfusion. 1997;37(6):573–6. 9191816 10.1046/j.1537-2995.1997.37697335150.x

[16] Roth A, Mercier A, Lepers C, Hoy D, Duituturaga S, Benyon E, et al. Concurrent outbreaks of dengue, chikungunya and Zika virus infections - an unprecedented epidemic wave of mosquito-borne viruses in the Pacific 2012–2014. Euro Surveill. 2014;19(41). 10.2807/1560-7917.es2014.19.41.20929 25345518

[17] Lanteri MC, Kleinman SH, Glynn SA, Musso D, Keith Hoots W, Custer BS, et al. Zika virus: a new threat to the safety of the blood supply with worldwide impact and implications. Transfusion. 2016;56(7):1907–14. 27282638 10.1111/trf.13677

[18] Barjas-Castro ML, Angerami RN, Cunha MS, Suzuki A, Nogueira JS, Rocco IM, et al. Probable transfusion-transmitted Zika virus in Brazil. Transfusion. 2016;56(7):1684–8. 27329551 10.1111/trf.13681

[19] Musso D, Nhan T, Robin E, Roche C, Bierlaire D, Zisou K, et al. Potential for Zika virus transmission through blood transfusion demonstrated during an outbreak in French Polynesia, November 2013 to February 2014. Euro Surveill. 2014;19(14). 10.2807/1560-7917.es2014.19.14.20761 24739982

[20] Motta IJ, Spencer BR, Cordeiro da Silva SG, Arruda MB, Dobbin JA, Gonzaga YB, et al. Evidence for transmission of Zika virus by platelet transfusion. N Engl J Med. 2016;375(11):1101–3. 27532622 10.1056/NEJMc1607262

[21] Mlakar J, Korva M, Tul N, Popović M, Poljšak-Prijatelj M, Mraz J, et al. Zika virus associated with microcephaly. N Engl J Med. 2016;374(10):951–8. 26862926 10.1056/NEJMoa1600651

[22] Tang H, Hammack C, Ogden SC, Wen Z, Qian X, Li Y, et al. Zika virus infects human cortical neural progenitors and attenuates their growth. Cell Stem Cell. 2016;18(5):587–90. 26952870 10.1016/j.stem.2016.02.016 PMC5299540

[23] Cao-Lormeau VM, Blake A, Mons S, Lastère S, Roche C, Vanhomwegen J, et al. Guillain-Barré Syndrome outbreak associated with Zika virus infection in French Polynesia: a case-control study. Lancet. 2016;387(10027):1531–9. 26948433 10.1016/S0140-6736(16)00562-6 PMC5444521

[24] Kuehnert MJ, Epstein JS. Assuring blood safety and availability: Zika virus, the latest emerging infectious disease battlefront. Transfusion. 2016;56(7):1669–72. 27389990 10.1111/trf.13673

[25] Musso D, Santa Maria F, Laughhunn A, Lanteri M, Stassinopoulos A, Aubry M. Pathogen inactivation of Zika, dengue and chikungunya viruses in all blood components. Vox Sang. 2017;112(Suppl. 1):64.28001314

[26] Giménez-Richarte Á, de Salazar MO, Arbona C, Giménez-Richarte MP, Collado M, Fernández PL, et al. Prevalence of chikungunya, dengue and Zika viruses in blood donors: a systematic literature review and meta-analysis. Blood Transfus. 2022;20(4):267–80. 34694219 10.2450/2021.0106-21 PMC9256504

[27] Giménez-Richarte Á, Ortiz de Salazar MI, Giménez-Richarte MP, Larrea L, Arbona C, Marco P, et al. Pathogen inactivation methods to prevent transfusion-transmissible arboviruses: a systematic review and meta-analysis. Trop Med Int Health. 2023;28(4):262–74. 10.1111/tmi.13863 36806816

[28] Marano G, Pupella S, Vaglio S, Liumbruno GM, Grazzini G. Zika virus and the never-ending story of emerging pathogens and transfusion medicine. Blood Transfus. 2016;14(2):95–100. 26674815 10.2450/2015.0066-15 PMC4786129

[29] Saa P, Chiu C, Grimm K, Yu G, Benjamin RJ, Corash L, et al. Acute Zika virus infection in an asymptomatic blood donor at the onset of the Puerto Rico epidemic. Transfusion. 2019;59(10):3164–70. 31407817 10.1111/trf.15484 PMC6785374

[30] Cardoso M, Ragan I, Hartson L, Goodrich RP. Emerging pathogen threats in transfusion medicine: improving safety and confidence with pathogen reduction technologies. Pathogens. 2023. 10.3390/pathogens12070911. 37513758 10.3390/pathogens12070911 PMC10383627

[31] Pagano MB, Rajbhandary S, Nunes E, Cohn CS. Transfusion services operations during the COVID-19 pandemic: results from AABB survey. Transfusion. 2020;60(11):2760–2. 33217023 10.1111/trf.15986 PMC7753805

[32] Kleinman S, Stassinopoulos A. Risks associated with red blood cell transfusions: potential benefits from application of pathogen inactivation. Transfusion. 2015;55(12):2983–3000. 26303806 10.1111/trf.13259 PMC7169855

[33] Kleinman SH, Lelie N, Busch MP. Infectivity of Human immunodeficiency virus-1, Hepatitis C virus, and Hepatitis B virus and risk of transmission by transfusion. Transfusion. 2009;49(11):2454–89. 19682345 10.1111/j.1537-2995.2009.02322.x

[34] Jacobs MR, Smith D, Heaton WA, Zantek ND, Good CE, Group PGDS. Detection of bacterial contamination in prestorage culture-negative apheresis platelets on day of issue with the Pan Genera Detection test. Transfusion. 2011;51(12):2573–82. 21883265 10.1111/j.1537-2995.2011.03308.x

[35] Corash L. Bacterial contamination of platelet components: potential solutions to prevent transfusion-related sepsis. Expert Rev Hematol. 2011;4(5):509–25. 21939419 10.1586/ehm.11.53

[36] Hong H, Xiao W, Lazarus HM, Good CE, Maitta RW, Jacobs MR. Detection of septic transfusion reactions to platelet transfusions by active and passive surveillance. Blood. 2016;127(4):496–502. 26598718 10.1182/blood-2015-07-655944

[37] Walther-Wenke G, Schrezenmeier H, Deitenbeck R, Geis G, Burkhart J, Hochsmann B, et al. Screening of platelet concentrates for bacterial contamination: spectrum of bacteria detected, proportion of transfused units, and clinical follow-up. Ann Hematol. 2010;89(1):83–91. 19484239 10.1007/s00277-009-0762-2

[38] Kopolovic I, Ostro J, Tsubota H, Lin Y, Cserti-Gazdewich CM, Messner HA, et al. A systematic review of transfusion-associated graft-versus-host disease. Blood. 2015;126(3):406–14. 25931584 10.1182/blood-2015-01-620872

[39] Mohammed H, Linnen JM, Muñoz-Jordán JL, Tomashek K, Foster G, Broulik AS, et al. Dengue virus in blood donations, Puerto Rico, 2005. Transfusion. 2008;48(7):1348–54. 18503611 10.1111/j.1537-2995.2008.01771.x

[40] Petersen LR, Tomashek KM, Biggerstaff BJ. Estimated prevalence of dengue viremia in Puerto Rican blood donations, 1995 through 2010. Transfusion. 2012;52(8):1647–51. 22304614 10.1111/j.1537-2995.2011.03529.x

[41] Snyder EL, Sekela ME, Welsby IJ, Toyoda Y, Alsammak M, Sodha NR, et al. Evaluation of the efficacy and safety of amustaline/glutathione pathogen-reduced RBCs in complex cardiac surgery: the Red Cell Pathogen Inactivation (ReCePI) study-protocol for a phase 3, randomized, controlled trial. Trials. 2023;24(1):799. 38082326 10.1186/s13063-023-07831-x PMC10712151

[42] Yee MEM, Josephson CD, Winkler AM, Webb J, Luban NLC, Leong T, et al. Hemoglobin A clearance in children with sickle cell anemia on chronic transfusion therapy. Transfusion. 2018;58(6):1363–71. 29664198 10.1111/trf.14610 PMC6021219

